# Temperature-Dependent Growth of *Geomyces destructans*, the Fungus That Causes Bat White-Nose Syndrome

**DOI:** 10.1371/journal.pone.0046280

**Published:** 2012-09-28

**Authors:** Michelle L. Verant, Justin G. Boyles, William Waldrep, Gudrun Wibbelt, David S. Blehert

**Affiliations:** 1 Department of Pathobiological Sciences, University of Wisconsin, Madison, Wisconsin, United States of America; 2 Department of Ecology and Evolutionary Biology, University of Tennessee, Knoxville, Tennessee, United States of America; 3 Department of Computer Science, University of Chicago, Chicago, Illinois, United States of America; 4 Leibniz Institute for Zoo and Wildlife Research, Berlin, Germany; 5 US Geological Survey – National Wildlife Health Center, Madison, Wisconsin, United States of America; Imperial College Faculty of Medicine, United Kingdom

## Abstract

White-nose syndrome (WNS) is an emergent disease estimated to have killed over five million North American bats. Caused by the psychrophilic fungus *Geomyces destructans*, WNS specifically affects bats during hibernation. We describe temperature-dependent growth performance and morphology for six independent isolates of *G. destructans* from North America and Europe. Thermal performance curves for all isolates displayed an intermediate peak with rapid decline in performance above the peak. Optimal temperatures for growth were between 12.5 and 15.8°C, and the upper critical temperature for growth was between 19.0 and 19.8°C. Growth rates varied across isolates, irrespective of geographic origin, and above 12°C all isolates displayed atypical morphology that may have implications for proliferation of the fungus. This study demonstrates that small variations in temperature, consistent with those inherent of bat hibernacula, affect growth performance and physiology of *G. destructans*, which may influence temperature-dependent progression and severity of WNS in wild bats.

## Introduction

White-nose syndrome (WNS) is an emergent disease of hibernating, insectivorous bats [1]. The causative agent, *Geomyces destructans* [2], [3], is a psychrophilic (cold-loving) fungus with active growth limited to cool environments such as those characteristic of underground bat hibernacula [4], [5]. WNS is characterized by invasive growth of *G. destructans* on the muzzle and wings of hibernating bats [6]. Since fungal infection consistent with WNS was first photo-documented among hibernating bats in a popular tourist cave in east-central New York during winter 2006–2007, over five million bats are estimated to have died from this disease (http://www.fws.gov/whitenosesyndrome/pdf/WNS_Mortality_2012_NR_FINAL.pdf). Population models predict that WNS could cause the little brown bat (*Myotis lucifugus*), once one of the most common bats across North America, to be extirpated from the northeastern United States by 2026 [7].

Following initial discovery and description of *G. destructans* in North America [1], the fungus was also identified on hibernating bats in twelve European countries [8], [9], [10], [11]. While infection of European bats by *G. destructans* has been documented to cause lesions diagnostic for WNS [12], infections have not been linked to unusual mortality events among wild European bats [13]. A recent laboratory study, however, demonstrated that isolates of *G. destructans* from Germany and New York were both lethal to an experimentally infected North American bat species, the little brown bat [3]. Reasons for the disparate effects of WNS on bat mortality between the two continents are unknown, and while intercontinental differences in bat physiology or behavior may be responsible, growth properties of individual fungal isolates, and/or environmental conditions may also play a contributing role [3], [14].

The psychrophilic nature of *G. destructans* likely restricts its fundamental niche, but detailed investigations of temperature-dependent growth of the fungus are lacking. Active growth of *G. destructans* has been previously reported between 0 and 20°C, but optimal temperature for growth remains unclear because previous measurements were conducted at low thermal resolution [1], [4]. Thus, the objectives of this study were to: 1) define the upper critical temperature for growth of *G. destructans*; 2) characterize temperature-dependent growth performance of the fungus from approximately 0°C to the upper critical temperature; 3) compare temperature-dependent growth performance for six isolates of *G. destructans* from North America and Europe; and 4) describe temperature-dependent variation in fungal morphology across a range of temperatures.

## Results

### Thermal optima and upper critical temperatures for growth

**Figure 1:**
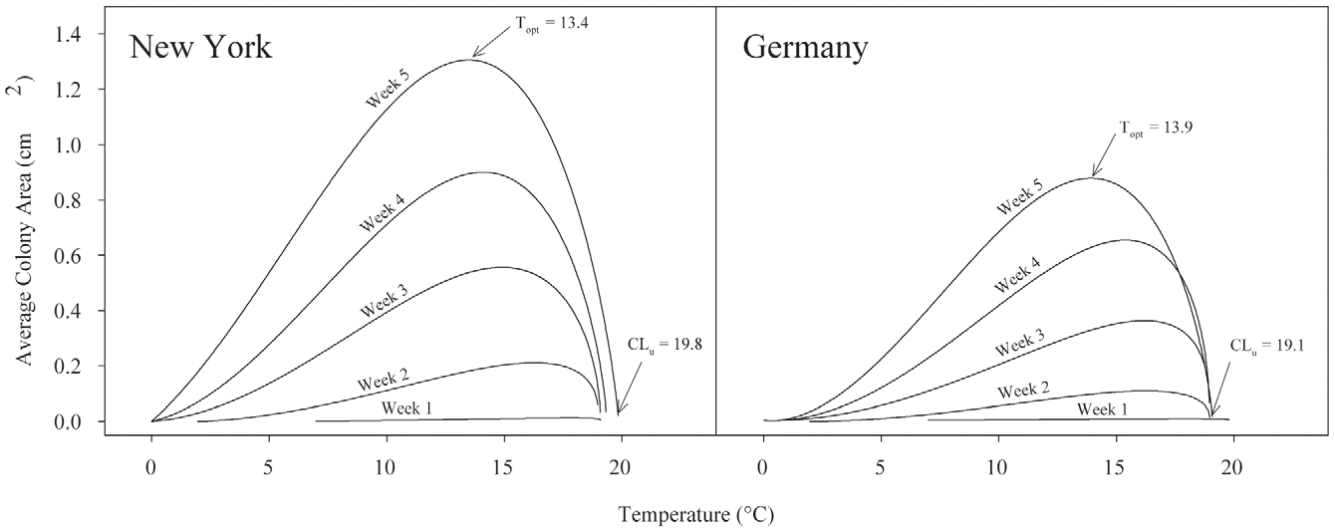
Weekly growth curves for two isolates of *Geomyces destructans*. In an initial experiment, two isolates of *G. destructans* (one from New York and one from Germany) exhibited differences in growth performance but had similar thermal optima and upper critical temperatures for growth. T_opt_ and upper critical temperatures (CL_u_) for growth at week 5 are marked on the graphs with arrows. For this figure, each curve is represented using a Brière2 function, although in some cases, other functions were equally parsimonious (Table S1). T_opt_ and CL_u_ in this figure represent the values specific to the Brière2 function shown in the graph; therefore T_opt_ does not match the weighted averages presented in Table 1. The isolates were grown on Sabouraud dextrose agar. Twenty-one replicate colonies of each were incubated across a range of nine temperatures from 0.8 to 21.4°C. The area of each expanding colony was measured weekly, for five weeks, and a growth curve was fit to each weekly dataset.

**Figure 2:**
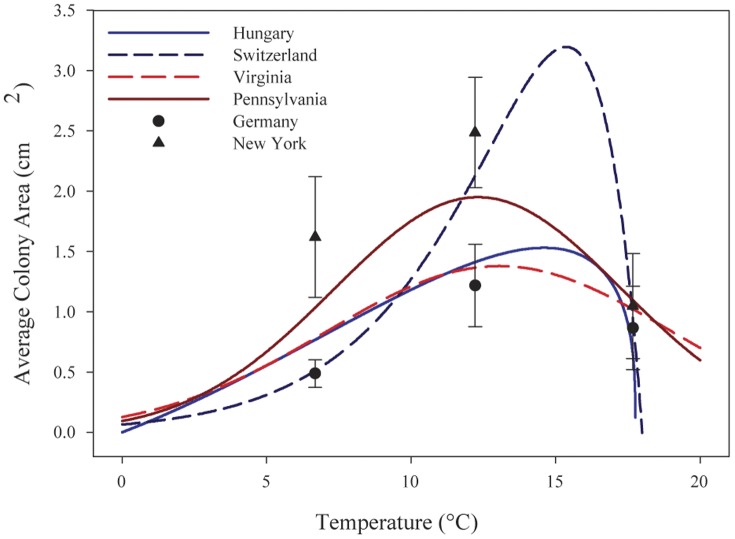
Five-week growth curves for four isolates of *Geomyces destructans*. In a follow-up experiment, differences in growth performance were confirmed among four additional isolates of *G. destructans*, two from North America and two from Europe. A consistent intercontinental trend in growth performance was not observed among the isolates. The isolates were grown on Sabouraud dextrose agar. Twenty-one replicate colonies of each isolate (Pennsylvania, Virginia, Hungary, and Switzerland) were incubated across a range of five temperatures from 1.9 to 17.7°C. The area of each expanding colony was measured after five weeks, and a growth curve was fit to each dataset. Each curve is represented using the best-fit function. For comparison to the weekly growth curve analysis (Fig. 1), 21 replicate colonies each of the isolates from New York and Germany were incubated with the other isolates at 6.7, 12.2, and 17.7°C. The area of each expanding colony was measured after five weeks, and descriptive data (mean ± SD) are represented. Isolates from New York and Germany grew proportionally faster at each of the temperatures used for this analysis than in the initial weekly analysis. Although the curve shapes for both analyses were consistent, the two analyses cannot be directly compared.

Thermal performance curves were generated for six independent isolates of *G. destructans* cultured from skin or hair of bats collected in New York, Pennsylvania, Virginia, Germany, Hungary, and Switzerland. Performance curves for all fungal isolates were characteristic in shape, with an intermediate peak at the thermal optimum for growth (T_opt_) and rapid decline in performance at temperatures above the peak (Figs. 1 and 2). Growth rates for each isolate increased slowly to a peak rate before decreasing to zero growth over a range of 3 to 5°C above T_opt_. There was variation in the best-fit function among isolates and weekly replicates, but skewed functions, particularly the Brière2 function, best described the growth data in most cases (Table S1). Following five weeks of incubation, T_opt_ varied across isolates, ranging from 12.5 to 13.8°C, with the exception of the Swiss isolate, which grew best at 15.8°C (Table 1). *Geomyces destructans* did not grow at 21.4°C, but growth was observed at 18.9°C, indicating the upper critical temperature is within this range. Estimates from functions that provided the means to calculate upper critical temperature for growth placed the value between 19.0 and 19.8°C. Eighty percent performance breadths (the range across which growth is >80% of maximal growth) for all isolates following five weeks of incubation were between 6.1 and 8.0°C (Table 1). The Swiss isolate had a narrower 80% performance breadth of 3.6°C (Table 1). In our initial experiment, which evaluated growth of two isolates each week for five weeks, performance breadth increased over time (Table 1) with symmetrical functions becoming reasonable fits to the data (i.e., ΔAICc<2) in the last week of sampling (Table S1). Variations in estimates of T_opt_ and performance breadth among subsets within each analysis were minimal, suggesting little intra-plate variation in colony growth. This small amount of variation in addition to the large number of randomizations led to small 84% CIs, and there was no overlap in CIs for any comparisons between replicates.

**Table 1:** Weighted averages of the thermal optima for growth (T_opt_) and 80% performance breadth for each isolate of *Geomyces destructans* grown in culture.

Isolate	T_opt_ (±84% CI)	Performance Breadth (±84% CI)
**New York**		
Week 1	16.98(0.008)	4.37(0.007)
Week 2	16.27(0.006)	5.06(0.006)
Week 3	14.92(0.007)	6.39(0.004)
Week 4	14.20(0.005)	6.67(0.004)
Week 5	13.05(0.004)	7.62(0.005)
**Germany**		
Week 1	16.87(0.012)	4.61(0.011)
Week 2	16.63(0.008)	5.20(0.007)
Week 3	16.20(0.003)	5.59(0.002)
Week 4	15.38(0.002)	6.03(0.001)
Week 5	13.79(0.003)	6.42(0.002)
**Pennsylvania**	12.45(0.002)	7.03(0.003)
**Virginia**	13.22(0.004)	7.99(0.006)
**Switzerland**	15.79(0.001)	3.64(0.003)
**Hungary**	13.96(0.005)	6.07(0.004)

Overlap in ±84% CI values indicates non-significant differences at the α = 0.05 level. Growth of isolates from Pennsylvania, Virginia, Switzerland, and Hungary were only measured at 5 weeks. All values are in degrees Celsius.

### Independent isolates exhibited differential growth performance

In an initial experiment comparing growth properties of two isolates of *G. destructans* previously shown in the laboratory to be lethal to little brown bats [3], an isolate from New York consistently grew faster than a German isolate across the entire range of temperatures examined (Fig. 1). Two independent best-fit functions were a better fit to the data (ΔAICc = 0) for the final week of measurements than was a single function describing the combined data (ΔAICc = 32.1), indicating substantial differences in temperature-dependent growth between these isolates.

The results of this initial experiment led to the hypothesis that isolates of *G. destructans* from North America grow faster than those from Europe. However, continent-dependent differences in growth performance were not verified by follow-up analysis in which growth performance of four additional isolates (two from North America and two from Europe) was measured (Fig. 2). Based upon this follow-up analysis, an isolate from Virginia exhibited slowest growth while the fastest growing isolate was from Switzerland. Four individual functions (ΔAICc = 0) collectively described the data better than one function for each continent (ΔAICc = 35.7), or than one function for all four isolates combined (ΔAICc = 60.8), further validating variation between isolates. However, with the exception of the Swiss isolate, small variations in growth parameters of the remaining five isolates (T_opt_ = 12.5 to 13.6°C; upper critical temperature for growth = 19.0 to 19.8°C; 80% performance breadths = 6.6 to 8.0°C) indicate that overall, growth performance was similar (Table 1).

### Morphology of Geomyces destructans varies with temperature

**Figure 3:**
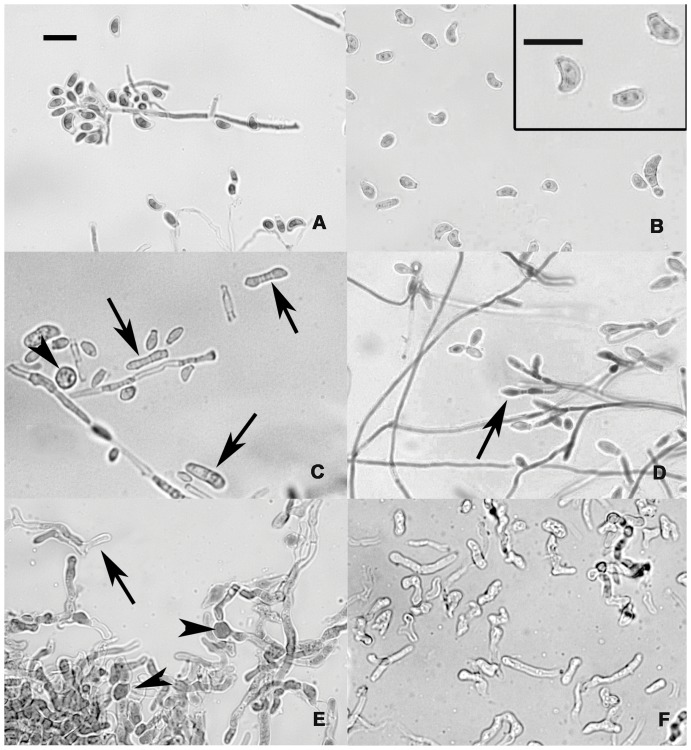
Morphology of *Geomyces destructans* varies with incubation temperature. (a) A characteristically branched conidiophore following growth at approximately 7°C. (b) Curved conidia typical of those produced following incubation at approximately 7°C. (c) Hyphae were thickened, fragmented into arthrospores (arrows), and produced chlamydospore-like structures (arrowhead) following incubation at approximately 12°C. (d) Conidia were primarily pyriform to globoid in shape and frequently formed short chains (arrow) following incubation at approximately 12°C. (e) At elevated temperatures (above 15°C), thickened, deformed hyphae showed evidence of degeneration, and hyphal tips exhibited branched antler-like morphology (arrow). Chlamydospore-like structures were also common (arrowhead). (f) Thick irregular hyphal fragments were produced by colonies grown at approximately 18°C; conidia were not observed. Scale bars, 10 µm.

We characterized morphology of hyphae and conidia produced by the North American type isolate of *G. destructans* following incubation at a range of temperatures (Fig. 3). Qualitative evaluations of the other isolates completed over the same temperature range revealed morphologies similar to those of the type isolate. Consistent with previous reports [4], when the type isolate was grown between approximately 0 and 7°C, it displayed colony morphology characteristic for *G. destructans*—white and smooth domed colonies with most developing light grey centers over time. Microscopically, hyphae were slim (average diameter = 1.5 µm) and yielded abundant, characteristically curved conidia from the tips of branched conidiophores (Fig. 3a, 3b). Atypical morphologies of hyphae and conidia were seen at approximately 12°C and above (Fig. 3c, 3d, 3e). At these high temperatures, hyphae were thicker (average diameter = 2.2 µm at ≈12°C; 3 µm at ≈18°C) and diffusely septate with differential sequestration of cellular material among segments. Conidia were mostly pyriform to globoid in shape, primarily produced in short chains (Fig. 3d; arrows), and released by rhexolytic dehiscence. Detachment of hyphal segments produced short arthrospore-like fragments (Fig. 3c, arrows), and dense single-cell structures resembling chlamydospores (Fig. 3c, 3e; arrowheads) were seen. At temperatures above approximately 15°C, hyphae were markedly deformed, and hyphal tips exhibited branched antler-like morphology (Fig. 3e; arrows). These changes became more pronounced at higher temperatures with complete loss of characteristic hyphal structure and curved conidia above approximately 18°C (Fig. 3d). Grossly, colonies grown above approximately 15°C were variably tan to dark brown, and colony surfaces were markedly creased. Gross and microscopic morphology of colonies grown at mid-range temperatures (approximately 7 to 12°C) included a mixture of these characteristics. Cultures of *G. destructans* maintained at approximately 7°C for up to one year did not exhibit morphological characteristics of cultures incubated at elevated temperatures.

## Discussion

We provide a detailed characterization of temperature-dependent growth of six isolates of *G. destructans* from North America and Europe and demonstrate inter-isolate variation in growth performance. Thermal performance curves generated for each isolate indicated thermal optima for growth between 12.5 and 15.8°C and an upper critical temperature for growth between 19.0 and 19.8°C. Although we observed differences in estimated growth curves among isolates, our analyses did not indicate that fungal isolates from North America and Europe have inherently different growth characteristics. Furthermore, a previously published experimental study demonstrated that isolates of *G. destructans* from North America and Europe were both lethal to a North American bat species [3]. Thus, observed inter-continental differences in WNS disease manifestation, progression, and mortality among wild bats do not likely result from continent-specific variations in growth performance of the pathogen.

The results of this study do, however, demonstrate that environmental conditions, specifically temperature, exert a strong influence on growth performance of *G. destructans* (Figs. 1 and 2). Consequently, differences in temperature at locations within underground sites occupied by hibernating bats may influence both progression and severity of WNS among infected bats and environmental persistence and transmission of the fungus. Hibernation temperatures preferred by bats vary considerably depending on species, hibernaculum characteristics, geographic location, duration of the hibernation season, and energetic condition of the animal [15], [16], [17]. For example, in a study of temperature preferences of hibernating bats in a limestone mine in Ohio [16], the majority of little brown bats hibernated at 7.2±2.6°C (mean ± SD), although some individuals hibernated across a wider range of temperatures (1.1 to 16.4°C) [15]. During hibernation, core body temperatures of bats are near or slightly above ambient temperature. Thus, a common body temperature of hibernating little brown bats of North America, approximately 7.2°C, is below the thermal optimum for growth of *G. destructans* (12.5 to 15.8°C).

Within the range of temperatures occupied by hibernating bats, variation is likely to be biologically important for individual animals infected by *G. destructans*. For example, in laboratory culture, the New York isolate of *G. destructans* exhibited a 93% reduction in growth performance from the upper (approximately 16°C) to lower (approximately 1°C) temperatures reported suitable for hibernation of little brown bats. From the mean hibernation temperature used by these bats (7.2°C), to one standard deviation below the mean (4.6°C) [16], our laboratory-based measurements indicate that growth performance of *G. destructans* is reduced by 40%. If these reductions in growth performance of the fungus are ultimately shown to yield similar reductions to the severity of fungal infection of hibernating bats, small reductions to hibernaculum temperatures (*e.g.*, 2 to 3°C) could serve to moderate disease progression in infected animals.

In addition to temperature-dependent variation in growth performance, we also observed changes in morphological characteristics of *G. destructans* when grown in culture at temperatures above approximately 12°C. Atypical characteristics were most apparent following incubation above approximately 15°C. These characteristics were not seen in cultures incubated at approximately 7°C for up to one year, indicating that observed morphological changes were temperature-, not age-dependent. Consistent with morphological changes observed in other fungal species grown at elevated temperatures [18], [19], [20], *G. destructans* exhibited increased septation and thickening of hyphae, altered conidial shapes, production of arthrospores, and formation of chlamydospore-like structures. Hyphal tips also exhibited branched antler-like morphology consistent with “favic chandeliers” characteristic of some pathogenic *Trichophyton* species [18]. Although *G. destructans* exhibited increased growth performance following incubation at elevated temperatures (12 to 15°C), the associated morphological changes we observed have been linked to stress response in other filamentous fungi [19], [20].

Morphological changes observed in cultures of *G. destructans* grown at higher temperatures may be biologically important for propagation and persistence of the fungus following transfer to warm environments. For example, under adverse conditions, arthrospores can become a primary means of fungal propagation [21], [22], and chlamydospores serve as desiccation-resistant, resting structures [19]. Consequently, if production of these fungal reproductive structures is promoted upon emergence of infected bats from hibernation, they may facilitate enhanced persistence of *G. destructans* within environments other than underground hibernacula. However, these altered reproductive structures do not contribute to fungal dispersal in as robust a manner as the abundant microconidia produced by *G. destructans* at cooler temperatures. Thus, although production of chlamydospores and arthrospores may facilitate survival of *G. destructans* outside of bat hibernacula, warmer temperatures either inside or outside of hibernacula may decrease the overall reproductive capacity of *G. destructans*. A better understanding of the basic biology and life cycle of *G. destructans* will be important for defining the epidemiology of WNS across the landscape.

Our laboratory-based investigations have revealed variation in physiological characteristics of *G. destructans* and highlight a need to determine how these differences may influence performance and dissemination of this fungus in natural environments. Similar phenotypic variation has been demonstrated among isolates of other fungi, including the amphibian pathogen, *Batrachochytrium dendrobatidis*, and the plant pathogen, *Phytophthora ramorum*, resulting in differential pathogenesis and risk for disease among susceptible host species [23], [24], [25]. Currently, little is known about how growth characteristics and performance of *G. destructans* on artificial medium compare to those of *G. destructans* on bats or as it persists or grows in association with environmental substrates. Likewise, it is unknown how natural body temperature patterns of bats during hibernation, which consist of long bouts of cold (torpor) interspersed by short periods of warmth (inter-torpor arousals), may affect growth performance and other characteristics of *G. destructans* as it colonizes their skin. Finally, little is known about microclimate selection of bats throughout the winter within a hibernaculum and how microclimate selection varies within a species across its geographic range. Considering the demonstrated variations in temperature-dependent growth of *G. destructans*, microclimate selection may be a critical factor in the epidemiology and manifestation of WNS. Thus, a more detailed understanding of how environmental variation within hibernacula influences physiology and growth performance of *G. destructans* across its geographic range may be key to understanding and managing the progression of WNS in hibernating bats.

## Materials and Methods

### Fungal isolates

Isolates of *G. destructans* used in this study were cultured during diagnostic investigations from skin or hair of bats collected in New York [4] (February, 2008; American Type Culture Collection number ATCC MYA-4855), Pennsylvania (March, 2009), Virginia (March, 2009), Germany [9] (March, 2009), Hungary [9] (March, 2009), and Switzerland [9] (April, 2009).

### Growth curves

Starter cultures for growth curves were initiated from frozen (−80°C) glycerol culture stocks by transfer to Sabouraud dextrose agar plates supplemented with gentamycin and chloramphenicol (SDA) and incubated at 7°C for approximately eight weeks. For each incubation temperature evaluated, starter culture material was transferred using a flame-sterilized inoculating needle to three evenly spaced locations on the surface of each of seven SDA plates, yielding 21 replicates of each isolate per temperature condition evaluated. This high level of colony replication was used to account for potential variability inherent in our method of inoculation. Each group of seven plates was sealed into a plastic container containing moist paper towels to maintain humidity. A temperature logger (iButton model 1923, Maxim Semiconductor, Dallas, Texas USA) programmed to record temperature every five minutes was maintained in one container per incubation temperature for the duration of each growth experiment.

### Weekly growth curve analysis

Isolates of *G. destructans* from New York and Germany were incubated for five weeks at nine different temperatures each. Mean incubation temperatures (calculated from recorded temperature logs) over the course of the five week growth period for the New York isolate were 0.8, 2.0, 4.6, 7.2, 11.9, 16.0, 17.6, 19.0, and 21.4°C; mean incubation temperatures for the German isolate were 0.8, 2.0, 4.6, 7.2, 11.9, 14.7, 17.6, 19.0, and 21.4°C. The environmental chambers maintained relatively constant temperatures with standard deviations ranging from 0.11 to 1.3°C. As we lacked sufficient incubators to test all temperatures simultaneously, a standardized growth curve analysis (21 colonies incubated at approximately 7°C) was generated in tandem with each group of temperatures evaluated to provide a means to compare non-simultaneous evaluations of growth. There were no significant differences in growth performance among three temporally independent growth curves generated from cultures incubated at approximately 7°C for either the New York isolate (*P* = 0.20) or the German isolate (*P* = 0.17).

### Five-week growth curve analysis

A second growth performance analysis was completed with isolates of *G. destructans* from Pennsylvania, Virginia, Hungary, and Switzerland. Mean incubation temperatures tested for these isolates were 1.9, 2.6 (Hungarian isolate only; this isolate was not grown at 1.9°C), 6.7, 12.2, 14.6, and 17.7°C. The environmental chambers maintained relatively constant temperatures with standard deviations ranging from 0.2 to 0.4°C. For comparative purposes, isolates of *G. destructans* from New York and Germany were grown in parallel and incubated at 6.7, 12.2, and 17.7°C. Using both frequentist and Bayesian analytical techniques, the New York and German isolates grew faster during the second experiment than the first (data not shown). This suggested that there was an undefined factor that affected overall growth rates, so we could not directly compare growth performance of these isolates from the two independent analyses. Thus, in the second analysis, we only directly compared the isolates for which we had growth measurements across the full range of temperatures (Pennsylvania, Virginia, Hungary, and Switzerland). However, this undefined factor affected only the absolute growth rates of each isolate and not the relative growth rates within an isolate; thus it should not have affected estimation of T_opt_ and performance breadth.

### Measurement of growth

For the weekly growth curve analysis, we determined temperature-dependent growth rates of the New York and the German isolates by measuring total surface area of visible growth (colony expansion) of each colony at weekly intervals for five weeks (referred to throughout as weekly replicates). Measurements of each colony were collected after visible growth was first apparent at the inoculation point. For the four-isolate analysis, colony area was analyzed at week five only. At each measurement interval, we captured digital gray-scale images of each culture plate using a FOTO/Analyst® Investigator CCD camera system (FOTODYNE Inc., Hartland, Wisconsin, USA) and ImageJ Version 1.34S (National Institutes of Health, Bethesda, Maryland, USA). A metric scale was included in each photograph for calibration. We calculated the surface area of each colony using image analysis software (Analyzing Digital Images Version 11, Museum of Science, Boston, Massachusetts, USA). A digital color mask was applied to all fungal colonies on a plate, and the area of masked pixels corresponding to each colony was measured in cm^2^. Gross colony morphology was also recorded.

### Analysis of growth curves

We used a curve-fitting procedure based on information criteria [26] to determine the function that best described temperature-dependent growth rates of *G. destructans* at each of the measurements for isolates in the weekly analysis and for the single measurement for each isolate in the five-week analysis. We used Akaike's Information Criterion adjusted for small sample size (AICc) [27] to compare relative fit of seven functions (Table S2): quadratic, Gaussian, modified Gaussian, Logan1, beta, performance, and Brière2 [26], [28], [29], [30], [31].

To account for possible plate effects in the initial experiment (*i.e.*, the weekly growth curve analysis; three colonies were grown on each plate), we analyzed randomly drawn subsets of data. To create each subset, we randomly selected the value of one colony from each plate at each temperature. We excluded any temperatures at which no colonies grew (*e.g.*, no colonies grew at 21.4°C, and no colonies grew at the coldest temperatures for the first two weeks) [32]; however, we used this information to constrain the values for critical minimum or maximum temperatures in the functions that specified those parameters (Logan1, beta, performance, and Brière2). If a colony was chosen that did not grow, it was not included and the sample size for that particular subset was reduced. If a colony never grew, we excluded that colony from sampling, and the sample sizes for all subsets were reduced. We made 10,000 such subsets of data for each isolate during each week.

We fit the seven curves to each subset using a custom-written script based on the LSQCURVEFIT function in MATLAB Version 7.12 (The MathWorks, Inc., Natick, Massachusetts, USA). For some random subsets, especially during the first two weeks of incubation when growth was poor, some of the resulting curves did not show the characteristic intermediate peak and were thus excluded. To do so, we removed any curve for which the second derivative was positive, or for which the first derivate was negative below the peak or positive above the peak. This ensured that only curves with the correct shape were included. We then averaged parameter and AICc values across the remaining subsets in each analysis. We chose the best-fit curve for each analysis based on average AICc values. Any curves with ΔAICc<2 were considered equally parsimonious. If less than 50% of subsets were fit properly by a given function, that function was removed from consideration as the best fit. We calculated two common descriptive characteristics for each subset, thermal optimum (T_opt_) and 80% performance breadth, and averaged the characteristics to get a value for each curve. We then averaged parameter estimates across the functions for which 50% of the subsets were good fits to calculate a single value for each characteristic in each isolate or weekly measurement. As per standard AIC technique [26], we weighted the parameter estimates by AICc weights so that better fitting models more strongly affected the average parameter estimate than did weaker models. The “optimal temperature” we estimated describes the “optimal temperature for maximum growth,” but we use T_opt_ for consistency with literature in the field. In context of our measurements, optimal temperature is only optimal for growth on SDA and not necessarily for reproduction or fitness. The upper critical temperatures for growth described for *G. destructans* are represented only for those functions that provide explicit estimates of this parameter and are therefore expressed as a range rather than an average.

To further evaluate initial differences in growth characteristics observed between isolates in each experiment, we conducted several additional analyses. For the weekly growth curve analysis, we determined if the two isolates following 35 days of incubation were best described using two separate curves or a single curve. For the five-week growth curve analysis, we determined if isolates were best described using separate curves, one curve for each continent, or one curve for all isolates combined. As observed plate effects in the initial analysis were minimal, we assumed equal plate effects across all replicates for this analysis, and we fit curves directly to the data without performing the randomization procedure on subsets of data as described above. The order of best fit models differed slightly using this technique from the order obtained by the randomization procedure, but parameter values for each curve were similar. We also compared the T_opt_ and performance breadths in each experiment [33]. As above, we used growth measurements collected after 35 days of incubation. We evaluated overlap of these characteristics in the 84% confidence intervals, which results in a Type I error rate of 0.05 [33], [34].

### Observation of morphology

Microscopic characteristics of each fungal isolate were examined following 8 weeks incubation at approximately 4, 12, 15, and 18°C. Prior to examination, fungal elements were stained using 1% Phloxine or lactophenol-cotton blue. Digital images were captured using a Leica DM2500 microscope (Leica Microsystems, GmbH, Wetzlar, Germany) with Retiga 2000R Fast 1394 camera (QImaging, Surrey, British Columbia, Canada). Average dimensions of hyphae and conidia at each incubation condition were calculated from measurements of ten randomly selected structures as determined using Image-Pro Analysis Version 6.0 (Media Cybernetics, Inc., Bethesda, Maryland, USA).

## Supporting Information

Table S1 Descriptive statistics and parameter values for best-fit functions of each isolate.
(DOCX)

Table S2 Mathematical formulas of the seven functions used for analyses.
(DOCX)
