# Supplementary material for: Temperature-Dependent Growth of Geomyces destructans, the Fungus That Causes Bat White-Nose Syndrome
Source: PLoS One. 2012 Sep 28;7(9):e46280. doi: 10.1371/journal.pone.0046280 (PMC3460873; doi:10.1371/journal.pone.0046280)
Supplement: Table S1 — Descriptive statistics and parameter values for best-fit functions of each isolate. (DOCX) [file pone.0046280.s001.docx]

**Table S1.** Descriptive statistics of each isolate and parameter values for the best-fit function and all other functions that were equally parsimonious to best-fit function. Parameter designations are provided in Table S2.

| Curve | AICc | | | ΔAICc | *w_i_* | T_opt_ (±84% CI) | Performance Breadth (±84% CI) | A | B | C | D | K |
| --- | --- | --- | --- | --- | --- | --- | --- | --- | --- | --- | --- | --- |
| **New York** | | | | | | | | | | | | |
| Week 1 |  | | |  |  |  |  |  |  |  |  |  |
| Logan1 | -292.46 | | | 0 | 0.60 | 16.78(0.005) | 4.13(0.0004) | 0.993959 | 0.328428 | 19.82695 | 3.044745 |  |
| Brière2 | -290.85 | | | 1.61 | 0.27 | 17.53(0.010) | 4.15(0.014) | 0.000052 | 3.791738 | 19.02728 | 6.055039 |  |
|  |  | | |  |  |  |  |  |  |  |  |  |
| Week 2 |  | | |  |  |  |  |  |  |  |  |  |
| Brière2 | -297.96 | | | 0 | 0.41 | 16.37(0.007) | 5.60(0.005) | 0.000578 | -0.31482 | 18.97639 | 3.280275 |  |
| Logan1 | -297.61 | | | 0.35 | 0.34 | 16.03(0.003) | 4.37(0.005) | 0.987951 | 0.312184 | 19.2476 | 3.21792 |  |
| Beta | -296.28 | | | 1.68 | 0.18 | 16.26(0.008) | 5.23(0.010) | -3.94855 | 19.02639 | 3.424702 | 0.464547 | 0.000639 |
|  |  | | |  |  |  |  |  |  |  |  |  |
| Week 3 |  | | |  |  |  |  |  |  |  |  |  |
| Brière2 | -214.11 | | | 0 | 0.60 | 14.81(0.007) | 6.47(0.003) | 0.001078 | -1.00124 | 19.08376 | 1.838801 |  |
|  |  | | |  |  |  |  |  |  |  |  |  |
| Week4 |  | | |  |  |  |  |  |  |  |  |  |
| Brière2 | -143.16 | | | 0 | 0.51 | 14.06(0.005) | 6.92(0.003) | 0.001252 | -1.83964 | 19.34675 | 1.424657 |  |
| Beta | -141.19 | | | 1.97 | 0.19 | 14.15(0.005) | 6.41(0.008) | -5.76409 | 19.55511 | 3.722674 | 0.977937 | 0.000711 |
|  |  | | |  |  |  |  |  |  |  |  |  |
| Week 5 |  | | |  |  |  |  |  |  |  |  |  |
| Brière2 | -87.66 | | | 0 | 0.30 | 13.45(0.005) | 7.66(0.004) | 0.001137 | -6.00583 | 19.83826 | 1.253516 |  |
| Gaussian | -87.48 | | | 0.17 | 0.27 | 12.49(0.003) | 7.06(0.004) | 1.322822 | 5.273962 | 12.4833 |  |  |
| Modified Gaussian | -86.30 | | | 1.36 | 0.15 | 12.13(0.023) | 8.71(0.009) | 1.269079 | 5.75469 | 2.907852 | 12.12683 |  |
|  |  | | |  |  |  |  |  |  |  |  |  |
| **Germany** | | | | | | | | | | | | |
| Week 1 |  | | |  |  |  |  |  |  |  |  |  |
| Logan1 | -372.57 | | | 0 | 0.47 | 16.97(0.003) | 4.13(0.0002) | 0.994062 | 0.328142 | 20.04689 | 3.047392 |  |
| Brière2 | -372.45 | | | 0.13 | 0.44 | 16.82(0.020) | 4.81(0.021) | 0.000022 | 4.980167 | 19.7217 | 4.549746 |  |
|  |  | | |  |  |  |  |  |  |  |  |  |
| Week 2 |  | | |  |  |  |  |  |  |  |  |  |
| Brière2 | -280.35 | | | 0 | 0.48 | 16.15(0.007) | 5.22(0.003) | 0.000331 | 3.77539 | 18.96649 | 2.570415 |  |
| Performance | -279.34 | | | 1.01 | 0.29 | 17.93(0.013) | 4.98(0.013) | 0.082862 | 4.732344 | 8.519778 | 18.98638 | 0.747979 |
| Beta | -278.49 | | | 1.87 | 0.19 | 16.03(0.006) | 5.67(0.007) | 4.134045 | 18.96147 | 1.441027 | 0.344905 | 0.002568 |
|  |  | | |  |  |  |  |  |  |  |  |  |
| Week 3 |  | | |  |  |  |  |  |  |  |  |  |
| Brière2 | -305.10 | | | 0 | 0.97 | 16.18(0.003) | 5.63(0.002) | 0.000945 | 0.799997 | 18.96 | 2.849088 |  |
|  |  | | |  |  |  |  |  |  |  |  |  |
| Week 4 |  | | |  |  |  |  |  |  |  |  |  |
| Brière2 | -260.68 | | | 0 | 1.00 | 15.38(0.002) | 6.03(0.0009) | 0.001477 | 0.797811 | 18.96107 | 2.089296 |  |
|  |  | | |  |  |  |  |  |  |  |  |  |
| Week 5 |  | | |  |  |  |  |  |  |  |  |  |
| Brière2 | -186.34 | | | 0 | 0.65 | 13.91(0.003) | 6.51(0.0007) | 0.001271 | 0.647883 | 19.08448 | 1.314379 |  |
| Beta | -184.39 | | | 1.95 | 0.24 | 13.88(0.003) | 6.52(0.003) | 0.735594 | 19.09319 | 1.925828 | 0.766784 | 0.001922 |
|  |  | | |  |  |  |  |  |  |  |  |  |
| **Hungary (5 Weeks)** |  | | |  |  |  |  |  |  |  |  |  |
| Brière2 | -50.17 | | | 0 | 0.41 | 14.53(0.006) | 6.64(0.004) | 0.002464 | -14.7105 | 17.7605 | 3.088594 |  |
| Gaussian | -49.15 | | | 1.02 | 0.25 | 12.09(0.002) | 6.22(0.003) | 1.542839 | 4.64546 | 12.08861 |  |  |
| Beta | -48.60 | | | 1.57 | 0.19 | 14.62(0.010) | 6.07(0.004) | -14.3553 | 17.91932 | 4.041016 | 0.484297 | 0.000078 |
|  |  | | |  |  |  |  |  |  |  |  |  |
| **Switzerland (5 Weeks)** | |  | |  |  |  |  |  |  |  |  |  |
| Logan1 | -60.75 | | | 0 | 0.60 | 15.36(0.001) | 3.58(0.006) | 0.998775 | 0.377283 | 17.99821 | 2.624721 |  |
| Beta | -58.96 | | | 1.79 | 0.25 | 16.52(0.001) | 3.46(0.004) | -6.06771 | 17.67006 | 4.028198 | 0.200586 | 0.000683 |
|  |  | | |  |  |  |  |  |  |  |  |  |
|  |  | | |  |  |  |  |  |  |  |  |  |
| **Virginia (5 Weeks)** |  | | |  |  |  |  |  |  |  |  |  |
| Gaussian | -47.88 | | | 0 | 0.76 | 13.05(0.004) | 7.99(0.006) | 1.378334 | 5.97116 | 13.04455 |  |  |
|  |  | | |  |  |  |  |  |  |  |  |  |
| **Pennsylvania (5 Weeks)** | | |  |  |  |  |  |  |  |  |  |  |
| Gaussian | -26.18 | | | 0 | 0.55 | 12.32(0.002) | 6.68(0.002) | 1.950963 | 4.994046 | 12.31033 |  |  |
