# Supplementary material for: Temperature-Dependent Growth of Geomyces destructans, the Fungus That Causes Bat White-Nose Syndrome
Source: PLoS One. 2012 Sep 28;7(9):e46280. doi: 10.1371/journal.pone.0046280 (PMC3460873; doi:10.1371/journal.pone.0046280)
Supplement: Table S2 — Mathematical formulas of the seven functions used for analyses. (DOCX) [file pone.0046280.s002.docx]

**Table S2.** Mathematical formulas utilized to fit each of the seven functions used in the analysis.

| **Function** | **Formula** |
| --- | --- |
| Gaussian | $ae^{\left. {-0.5\times\left( \frac{x-c}{b} \right)}^{2} \right.}$ |
| Modified Gaussian | $ae^{\left. {-0.5\times abs\left( \frac{x-d}{b} \right)}^{c} \right.}$ |
| Beta | $k\left. \left( x-a \right)^{c}\left( b-x \right)^{d} \right.$ |
| Brière2 | $ax\left. \left( x-b \right)(c-x) \right.^{\frac{1}{d}}$ |
| Performance | $k\left. \left( 1-e^{\left. -a\left( x-b \right) \right.} \right)\left( 1-e^{\left. c\left( x-d \right) \right.} \right) \right.$ |
| Logan1 | $a\left( e^{bx}-e^{\left. bc-\left. \frac{c-x}{d} \right. \right.} \right)$ |
| Quadratic | $a+bx+cx^{2}$ |
